# Microbial Community Structure and Function Indicate the Severity of Chromium Contamination of the Yellow River

**DOI:** 10.3389/fmicb.2018.00038

**Published:** 2018-01-25

**Authors:** Yaxin Pei, Zhengsheng Yu, Jing Ji, Aman Khan, Xiangkai Li

**Affiliations:** Ministry of Education Key Laboratory of Cell Activities and Stress Adaptations, School of Life Sciences, Lanzhou University, Lanzhou, China

**Keywords:** the Yellow River, Cr, microbial indicator species, MiSeq sequencing, qRT-PCR, Cr (VI) reduction test

## Abstract

The Yellow River is the most important water resource in northern China. In the recent past, heavy metal contamination has become severe due to industrial processes and other anthropogenic activities. In this study, riparian soil samples with varying levels of chromium (Cr) pollution severity were collected along the Gansu industrial reach of the Yellow River, including samples from uncontaminated sites (XC, XGU), slightly contaminated sites (LJX, XGD), and heavily contaminated sites (CG, XG). The Cr concentrations of these samples varied from 83.83 mg⋅kg^-1^ (XGU) to 506.58 mg⋅kg^-1^ (XG). The chromate [Cr (VI)] reducing ability in the soils collected in this study followed the sequence of the heavily contaminated > slightly contaminated > the un-contaminated. Common Cr remediation genes *chrA* and *yieF* were detected in the XG and CG samples. qRT-PCR results showed that the expression of *chrA* was up-regulated four and threefold in XG and CG samples, respectively, whereas the expression of *yieF* was up-regulated 66- and 7-fold in the same samples after 30 min treatment with Cr (VI). The copy numbers of *chrA* and *yieF* didn’t change after 35 days incubation with Cr (VI). The microbial communities in the Cr contaminated sampling sites were different from those in the uncontaminated samples. Especially, the relative abundances of *Firmicutes* and *Bacteroidetes* were higher while *Actinobacteria* was lower in the contaminated group than uncontaminated group. Further, potential indicator species, related to Cr such as Cr-remediation genera (*Geobacter, PSB-M-3, Flavobacterium*, and *Methanosarcina*); the Cr-sensitive genera (*Skermanella, Iamia, Arthrobacter*, and *Candidatus Nitrososphaera*) were also identified. These data revealed that Cr shifted microbial composition and function. Further, Cr (VI) reducing ability could be related with the expression of Cr remediation genes.

## Introduction

River contaminants, especially heavy metals, because of their high toxicity, abundance, and persistent properties, can damage aquatic ecosystems (Varol, 2011; Liao et al., 2016). The Yellow River, the second largest river in China and the sixth largest river in the world, irrigates 15% of the agricultural land, supports a population of 107 million, and contributes 9% to China’s GDP (Hu et al., 2015). The Yellow River is an important water source in arid northern China and suffers the continually increasing environmental pressures from large amounts of pollutants, especially heavy metals, since it receives billions of tons of sewage annually resulting from anthropogenic activities (Yuan et al., 2012; Liu et al., 2015). A large number of water-intensive industries such as petrochemical industries, mining, and animal husbandry are located in cities along the Gansu industrial reach of the Yellow River and are strongly dependent on the Yellow River for their water demands (Ma et al., 2016). However, studies about heavy metals toxicity, mobility, and bioavailability along Gansu industrial reach of the Yellow River are still scarce.

Several studies have shown riparian soils, due to a variety of processes such as adsorption, biological uptake, and sedimentation to reflect the heavy metal contamination conditions of aquatic ecosystems. For example, heavy metal contamination conditions were evaluated for the Pearl River estuary by collecting riparian soil samples (Bai et al., 2011b). In another study, the effects of heavy metals on microbial communities were assessed using riparian soils around a settling pond for mine drainage treatment (Fan et al., 2016). Numerous studies have revealed that microbes are much more sensitive to heavy metals than plants in the same area (Giller et al., 2009; Sun et al., 2012). Thus, microbial community structure could also be a good indicator for revealing the severity of heavy metal contamination. For instance, *Firmicutes* and *Bacteroidetes* are the main phyla in the Cr contaminated environments (Miao et al., 2015; Zhang et al., 2017), whereas another study showed that influent zones, which possess higher heavy metal concentrations, contained more *Firmicutes, Bacteroidetes*, and *Actinobacteria*in in comparison to upstream, downstream, and effluent zones did (Fan et al., 2016). Microorganisms exposed to strong selective pressures from heavy metal contaminated environments can process corresponding function for the ecosystem (Epelde et al., 2015). This has resulted in the evolution of heavy metal resistance mechanisms, including not only the structural changes of microbial communities (Fan et al., 2016), but also transfer of heavy metal resistance genes to other community members by transposons or plasmids and expression level changes of such genes (He et al., 2011; Epelde et al., 2015). Thus, the phenotype of microbial communities can also reflect the severity of the pollution. However, studies about the correlation between phenotypic function and composition of microbial community have not been reported along Gansu industrial reach of the Yellow River.

Chromium is a common pollutant in the river (Zhang et al., 2009; Bai et al., 2011a). Cr (VI) and Cr (III) are the stable forms of Cr commonly found in nature. Highly toxic Cr (VI) can be reduced to Cr (III) by microbes (Pradhan et al., 2016). Cr concentration in the sediments of Xigu area and the isolated Cr-remediation bacteria have been reported (Liu et al., 2009; Huang et al., 2016). Thus, this study is focused on the Cr contamination in Gansu industrial reach of the Yellow River. In this study, riparian soil samples were collected from six sites along Gansu industrial reach of the Yellow River with a contamination gradient of Cr pollution. The main objectives of this study were: (1) to study the effect of environmental factors on the microbial diversity and composition along the Gansu industrial reach of the Yellow River; (2) to understand the functional genes of the microbial communities involved in the Cr (VI) remediating process; and (3) to determine which microbial taxa can be used as indicators for revealing the Cr contamination level in Gansu industrial reach of the Yellow River aquatic ecosystem.

## Materials and Methods

### Study Sites and Sampling

**FIGURE 1:**
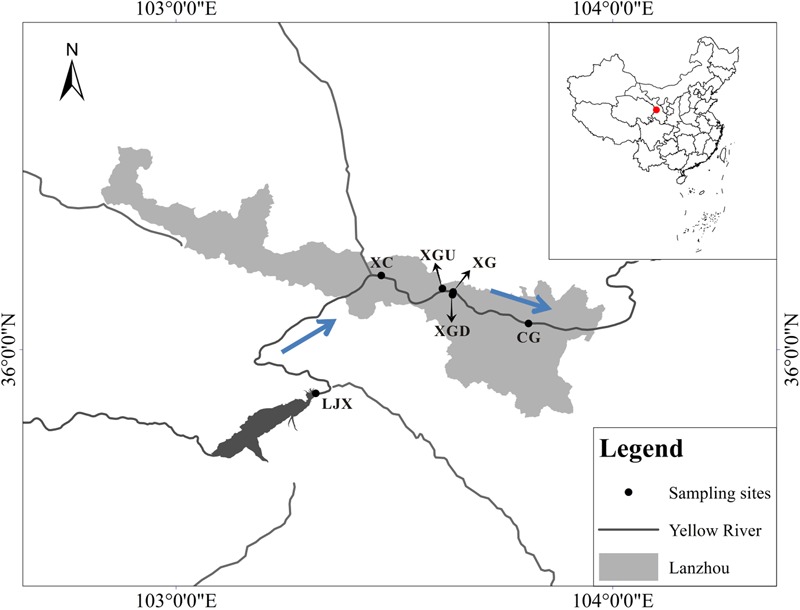
Location of sampling sites along the Gansu industrial reach of the Yellow River. Three categories are uncontaminated regions – Xincheng district (XC) and upstream of Xigu district (XGU), slightly contaminated regions - downstream of Xigu district (XGD) and the Liujiaxia reservoir (LJX) and heavily contaminated regions - Xigu district (XG) and Chengguan district (CG). The arrows indicated the flowing direction of the Yellow River.

Riparian soil samples were collected from six different sites along the Gansu industrial reach of the Yellow River in August of 2014 (**Figure 1**). The Xigu district (XG) in Lanzhou, because of its industrial prosperity, has long been reported as contaminated by heavy metals such as Cu, Zn, Pb, and Cr (Yu et al., 2016). The Chengguan district (CG) has also been polluted for over 50 years as a consequence of the discharge of domestic sewage discharge (Luo et al., 2011). The Liujiaxia reservoir (LJX) was found to be at a middle level of contamination in 2012 from analyzing the characteristics of algal communities (Sang et al., 2012). The downstream of Xigu district (XGD) sample was collected downstream of XG. It was polluted, but not as severely as the XG sample and the pollution could be a result of the recent industrial expansion. Moreover, no contamination was reported in Xincheng district (XC) and upstream of Xigu district (XGU) because of the fewer factories and population. Five spatially independent subsamples (2 cm in diameter, 0–15 cm in depth) were collected from each site along the bank of the Yellow River (10 m intervals) and combined to act as one sample. Three of these combined riparian soil samples were collected independently at each sampling site. The riparian soil samples were transferred to the lab on ice and then each sample was weighed and divided into three subsamples: the first set of subsamples was used for RNA extraction and immediately incubation with Cr (VI); the second was kept at room temperature for determining physicochemical properties; and the third was kept at -80°C for DNA extraction and downstream analyses.

### Physicochemical Detection of Riparian Soils

Soils were air-dried until their weights were stable and then sieved (<1 mm) for the following processes. Soil moisture was measured with thermo-gravimetric method (Al-Kayssi, 2002). The pH was measured with a pH meter after incubating a 1:5 mixture of riparian soil to 1 M KCL for 2 h (Wu et al., 2010). N, P, soil organic matter (SOM), the total concentration of metals (K, Cu, Zn, Cr, and Mn), and the salt extractable fractions (SK, SCu, SZn, SCr, and SMn) of metals were measured as previously described (Havlin and Westfall, 1985; Akcay et al., 2003; Sitters et al., 2013; Yu et al., 2016). Heavy metal concentrations that exceeded the natural background values were regarded as polluted sites (Bao et al., 2009).

### Illumina MiSeq Sequencing of 16S rDNA

DNA was extracted using a soil DNA Extraction Kit (MO BIO, United States, 12888-50). Each site was processed in triplicate. DNA concentration and quality were determined using a NanoDrop2000 Spectrophotometer (Thermo Scientific, United States). Totally, 18 extracted genomic DNA samples were sent to the Chengdu Institute of Biology for high-throughput sequencing using primers of 16s rRNA gene [515F (5′-GTGCCAGCMGCCGCGGTAA-3′) and 909R (5′-CCCCGYCAATTCMTTTRAGT-3′)] (Wu et al., 2016). The original pyrosequencing data are available from the MG-RAST database (accession no. 4750235.3 – 4750252.3).

### Cr (VI) Reduction Test of the Soil Microbial Community

To detect the Cr (VI) reduction ability of the microbial community in different riparian soil samples, for each fresh riparian soil sample, 10 g of the sample was added to a 50 mL plastic tube spiked with 20 mL 0.85% NaCl. An additional tube for each sample was autoclaved (110 kPa, 121°C, 1 h) for complete sterilization and regarded as a control because a previous study reported that the Cr (VI) reduction ability of soil samples was not affected by autoclave sterilization but the microbes in the soil were (Xiao et al., 2014). K_2_Cr_2_O_7_ was added to the tubes for a final concentration of 0.05 mM. The sterile and non-sterile riparian soil samples were incubated at 37°C for 35 days. Additionally, more K_2_Cr_2_O_7_ was added to the initial concentration once the yellow color of the initial K_2_Cr_2_O_7_ solution faded completely in riparian soil samples. Riparian soil moisture was maintained at a constant level by periodic watering. Each sample was processed in triplicate. Finally, detection of Cr (VI) concentrations was carried out via spectrophotometry using the Cr (VI)-specified reagent *S*-diphenylcarbazide (DPC) (Pattanapipitpaisal et al., 2001). Cr (VI) reduction ability was evaluated using the following formula:

Cr (VI) reduction ability (%) = [(Cr_A_ - Cr_B_)/Cr_A_] × 100

where Cr_A_ is the total Cr consumption amount in the non-sterile group, including reduced and adsorbed Cr (VI) amount; and Cr_B_ is the total adsorbed Cr (VI) amount in the sterile group.

### Real-Time Quantitative RT-PCR (qRT-PCR)

Two Cr remediation related genes, *chrA* (chromate transporter) and *yieF* (chromate reductase) were detected using genomic DNA as the template in PCR with Ex Taq^TM^ (TaKaRa, Japan, RR001A) following the manufacturer’s instructions. Riparian soil samples were treated with 0.85% NaCl containing 0.5 mM K_2_Cr_2_O_7_ or 0.85% NaCl only for 30 min. Subsequently, total RNA was extracted from the riparian soil samples above using an RNA PowerSoil^®^ Total RNA Isolation Kit (MO BIO, United States, 12866-25). Any remaining genomic DNA was removed via an RTS DNase TM Kit (MO BIO, United States, 15200-50). Then, first-strand cDNA synthesis was completed using a PrimeScript^TM^ RT Reagent Kit (TaKaRa, Japan, RR047A) according to the manufacturer’s protocols. Expression levels of *chrA* and *yieF* after 30 min Cr (VI) treatment, copy numbers changes of *chrA* and *yieF* after 35 days of chromate incubation, and microbial biomass were assessed via real-time PCR. Reactions were performed on CFX96^TM^ Real-Time PCR Detection System (Bio-Rad) according to the manufacturer’s instructions (Wu et al., 2014). All genes were analyzed in triplicate. The primer sequences of the target genes and the 16S rRNA gene are listed in Supplementary Table S1.

### Statistical Analysis

Data were expressed as the mean ± SD. Soil physicochemical properties, microbial diversity indices, Cr (VI) reduction abilities, and RT-PCR results were examined by Tukey’s test. We used Spearman’s rank correlation coefficient to determine whether there was a significant correlation between different environmental variables or between the relative abundances of dominant microbes and the environmental variables. The statistical analyses were performed in SPSS 16.0 for Windows. Principal coordinate analysis (PCoA), Canonical correspondence analysis (CCA), and Variation partition analysis (VPA) were performed using R version 2.15.2 as described before (R Core Team, 2015; Yu et al., 2016).

## Results

### Riparian Soil Physicochemical Properties

The physicochemical properties of the soil samples collected from the six sites were measured. These included soil texture (Supplementary Figure S1), pH, water content, the concentrations of N, P, K, Cu, Zn, Cr, and Mn; and SOM (**Table 1**). The pH values of the soil samples varied slightly, from 7.64 to 8.28. The XG samples contain higher amount of N and K, while the P content was almost the same across all site samples. The Cr concentrations ranged from 83.83 to 506.58 mg kg^-1^. Based on the Cr contamination states, the six sites were divided into three different categories: (i) uncontaminated regions, including XC and XGU, where the Cr concentration was less than the natural background (90 mg kg^-1^); (ii) slightly contaminated regions where the Cr concentration is greater than the natural background (90 mg kg^-1^), but less than the limited standard of Chinese Soil Quality Criterion (250 mg kg^-1^). These included regions such as XGD and the LJX; and (iii) heavily contaminated regions, including XG and CG, where the Cr concentration exceed the limited standard of Chinese Soil Quality Criterion (250 mg kg^-1^) (**Table 1**). On the other hand, the pollution conditions for Cu, Zn, and Mn were not as severe as Cr and only showed contamination at the XG site. The salt extractable fraction of metals like K, Cu, Zn, and Cr were detected in higher concentrations in XG samples in comparison to other sites the concentration of Mn was higher in CG samples.

**Table 1:** Physicochemical properties of the sites’ soil samples.

	Different sites’ soil samples					
Variables	XC	XGU	LJX	XGD	CG	XG
pH	8.03 ± 0.05^bc^	7.64 ± 0.05^e^	8.28 ± 0.03^a^	7.90 ± 0.01^d^	8.15 ± 0.06^b^	7.98 ± 0.06^cd^
Water content (%)	9.46 ± 0.46^d^	9.75 ± 0.86^d^	25.38 ± 0.90^c^	30.27 ± 0.65^b^	33.98 ± 1.30^ab^	37.61 ± 3.88^a^
N (mg ⋅ kg^-1^)	7296.23 ± 326.87^bcd^	9723.15 ± 1859.78^b^	5957.70 ± 1206.44^cd^	5383.55 ± 325.96^d^	9033.46 ± 330.46^bc^	14397.08 ± 1525.51^a^
P (mg ⋅ kg^-1^)	879.18 ± 62.79^a^	688.24 ± 87.27^a^	746.06 ± 82.46^a^	685.86 ± 30.15^a^	1081.08 ± 285.85^a^	885.68 ± 178.13^a^
K (mg ⋅ kg^-1^)	8235.00 ± 103.76^bc^	8006.33 ± 177.43^c^	8442.00 ± 55.68^bc^	8676.67 ± 227.73^b^	8222.00 ± 166.44^bc^	18220.67 ± 258.81^a^
Cu (mg ⋅ kg^-1^)	3.63 ± 0.53^c^	9.19 ± 0.68^b^	8.69 ± 0.34^b^	8.12 ± 0.62^bc^	10.50 ± 0.55^b^	28.58 ± 3.83^a^
Zn (mg ⋅ kg^-1^)	66.57 ± 9.27^b^	57.64 ± 6.95^b^	65.17 ± 0.55^b^	66.08 ± 4.77^b^	62.28 ± 6.95^b^	342.55 ± 4.68^a^
Cr (mg ⋅ kg^-1^)	87.35 ± 27.08^c^	83.83 ± 6.02^c^	116.37 ± 13.75^c^	135.08 ± 12.47^c^	239.02 ± 34.93^b^	506.58 ± 13.79^a^
Mn (mg ⋅ kg^-1^)	5685.67 ± 230.53^bc^	5056.67 ± 246.58^d^	5456.00 ± 35.51^c^	5756.00 ± 26.85^bc^	5895.33 ± 14.50^b^	10753.33 ± 45.09^a^
SK (mg ⋅ kg^-1^)	37.06 ± 0.47^d^	40.03 ± 0.89^ab^	37.99 ± 0.25^cd^	39.91 ± 1.05^bc^	38.64 ± 0.78^bcd^	41.91 ± 0.60^a^
SCu (mg ⋅ kg^-1^)	0.02 ± 0.00^d^	0.05 ± 0.00^bc^	0.04 ± 0.00^bc^	0.04 ± 0.00^c^	0.05 ± 0.00^b^	0.07 ± 0.01^a^
SZn (mg ⋅ kg^-1^)	0.30 ± 0.04^b^	0.29 ± 0.03^b^	0.29 ± 0.00^b^	0.30 ± 0.02^b^	0.29 ± 0.03^b^	0.79 ± 0.01^a^
SCr (mg ⋅ kg^-1^)	0.39 ± 0.01^d^	0.43 ± 0.02^d^	0.52 ± 0.01^c^	0.62 ± 0.02^b^	1.12 ± 0.02^a^	1.18 ± 0.06^a^
SMn (mg ⋅ kg^-1^)	25.59 ± 1.04^bc^	25.28 ± 1.23^bc^	24.55 ± 0.16^c^	26.48 ± 0.12^ab^	27.71 ± 0.07^a^	24.73 ± 0.10^bc^
SOM (mg ⋅ g^-1^)	1.68 ± 0.16^b^	1.56 ± 0.17^b^	2.76 ± 0.10^b^	3.32 ± 0.26^b^	5.36 ± 0.65^b^	29.57 ± 6.13^a^

*All data are presented as mean ± standard deviation (*n* = 3). Values with different letters in a row mean significant differences at *p* < 0.05 as determined by Turkey’s test.*

### Cr (VI) Reduction Test of the Soil Microbial Community

**FIGURE 2:**
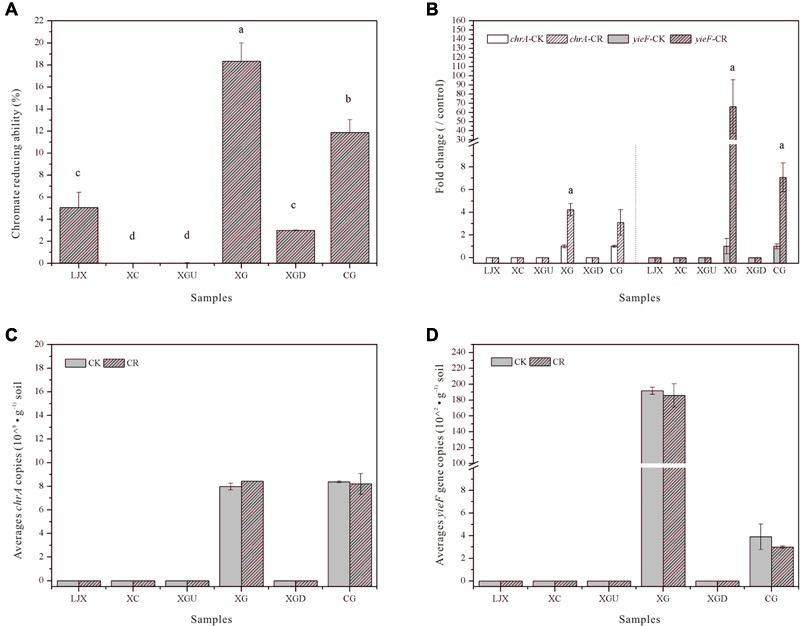
**(A)** Cr (VI) reduction ability of soil samples from the six sites. **(B)** Expression of mRNA of *chrA* and *yieF* in riparian soil samples treated with 0.85% NaCl containing 0.5 mM K_2_Cr_2_O_7_ for 30 min. The control group was treated with 0.85% NaCl only (CK). Mean expression in the treated group is shown as fold change compared to the mean expression in the control group, which has been ascribed an arbitrary value of 1. Significant difference at the *p* < 0.05 level is signified by ^a^. **(C)** Copies of *chrA* in riparian soil samples incubated with 0.85% NaCl only (CK) and 0.85% NaCl containing 0.05 mM K_2_Cr_2_O_7_ (CR) for 35 days. **(D)** Copies of *yieF* in the riparian soil sample incubated with 0.85% NaCl only (CK) and 0.85% NaCl containing 0.05 mM K_2_Cr_2_O_7_ (CR) for 35 days. Values with different letters mean significant differences at *p* < 0.05, as determined by Tukey’s test.

To evaluate the severity of Cr in Gansu industrial reach of the Yellow River, the Cr (VI) reduction abilities of the riparian soil microbial communities from different samples were measured (**Figure 2A**). No Cr (VI) reduction was observed for the XC and XGU samples which may be due to the low Cr stress in these sites. The heavily contaminated group (XG, CG) exhibited the highest Cr (VI) reduction ability, whereas the slightly contaminated group (LJX, XGD) had moderate Cr (VI) reduction ability. The Cr (VI) reduction ability of the uncontaminated, slightly contaminated, and heavily contaminated groups are probably associated with the severity of Cr contamination. Additionally, Spearman’s correlation analysis showed that Cr (VI) reduction ability did increase with Cr concentrations (Supplementary Table S3). Therefore, microbial function could indicate the severity of Cr contamination.

### *chrA* and *yieF* Related to Cr (VI) Remediation

Two common Cr remediation genes *chrA* (chromate transporter) and *yieF* (chromate reductase) were chosen to explain the different Cr (VI) reduction abilities among the samples. After treatment with Cr (VI) for 30 min, RNA was extracted from the riparian soil samples and the qRT-PCR results showed that the expression of *chrA* was up-regulated four and threefold in XG and CG samples, respectively, whereas the expression of *yieF* was up-regulated 66- and 7-fold in the same samples (**Figure 2B**). Further, copies of *chrA* and *yieF* did not change after being incubated with Cr (VI) in lab for 35 days (**Figures 2C,D**). *chrA* and *yieF* were not detected in uncontaminated and slightly contaminated groups because Cr (VI) contamination of those sites was not as severe as that of CG and XG sites (**Figures 2B–D**). This result not only indicated that the Cr (VI) reduction ability of the CG and XG soil samples might be related to the expression of Cr (VI) remediation genes but also further implied that Cr may only influenced the microbial community functions at the transcriptional level nor copy numbers of genes.

### Microbial Community Grouping Based on the Contamination Situations

MiSeq sequencing data of the 16S rRNA gene showed that 9080 qualified reads were obtained from each sample and 18,097 operational taxonomic units were obtained based on 3% dissimilarity (Supplementary Figure S2). Microbial diversity indices of each sample were in close range except for slightly lower richness and diversity of the LJX samples (Supplementary Table S2).

**FIGURE 3:**
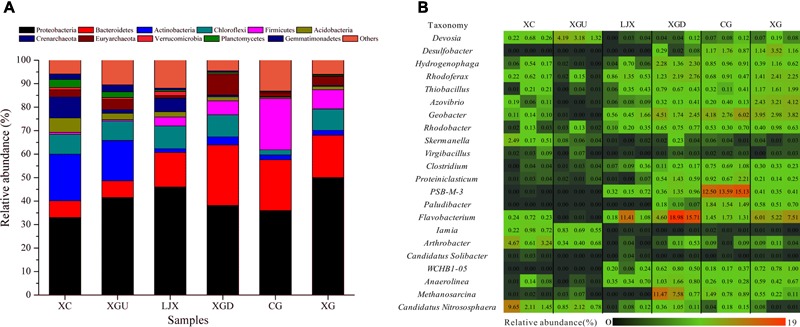
**(A)** Phylogenetic distributions of the phyla (% of total reads) present in different riparian soil samples. **(B)** Relative abundances of the 22 most dominant microbial genera for the six riparian sites.

For these sites, 66 different phyla were observed. At least 87% of total reads were affiliated with bacterial phyla and 4.48% of total reads were assigned to archaeal phyla. *Proteobacteria* (33.03–49.99%) was the most abundant phylum for all six sites, followed by *Bacteroidetes* (7.07–25.73%), *Actinobacteria* (1.59–19.89%), *Chloroflexi* (2.14–9.69%), *Firmicutes* (0.58–21.85%), and *Acidobacteria* (0.57–6.25%). In this study, these six phyla accounted for more than 67% of the total bacteria. The archaea *Crenarchaeota* (0.23–8.98%) and *Euryarchaeota* (1.05–8.73%) were also dominant (**Figure 3A**).

The distributions of each phylum at the six riparian sites (**Figure 3A**) imply that the dominant species of the uncontaminated group (XC and XGU) were remarkably different from that of the contaminated group (LJX, XGD, CG, and XG). The most abundant phyla associated with the contaminated group (LJX, XGD, CG, and XG) were *Proteobacteria, Bacteroidetes*, and *Firmicutes*. In comparison, *Firmicutes* and *Bacteroidetes* content in the contaminated group was higher than those in the uncontaminated group, whereas *Proteobacteria* did not differ much between the two groups. In contrast, *Actinobacteria* was more prevalent in the uncontaminated group than in the contaminated group. Almost all samples contained similar amounts of *Chloroflexi* except for the CG site.

Analysis of microbial composition at the genus level provided additional information on microbial adaption in response to environmental variations. Among all riparian soil samples, 1469 genera were detected and 22 of the most dominant genera showed differences among samples (**Figure 3B**). In the contaminated group (LJX, XGD, CG, and XG), the microbial communities were dominated by *Desulfobacter, Hydrogenophaga, Rhodoferax, Thiobacillus, Azovibrio, Geobacter, Clostridium, Proteiniclasticum, PSB-M-3, Flavobacterium, WCHB1-05* and *Anaerolinea*, and *Methanosarcina*. All these genera belong to *Proteobacteria, Firmicutes, Bacteroidetes*, and *Chloroflexi* which were the most abundant phyla in the contaminated group (**Figure 3B**). In contrast, the uncontaminated group consisted of completely different dominant bacteria including *Skermanella, Iamia, Arthrobacter*, and *Candidatus Nitrososphaera* which are affiliated to the *Proteobacteria, Actinobacteria*, and *Crenarchaeota*.

**FIGURE 4:**
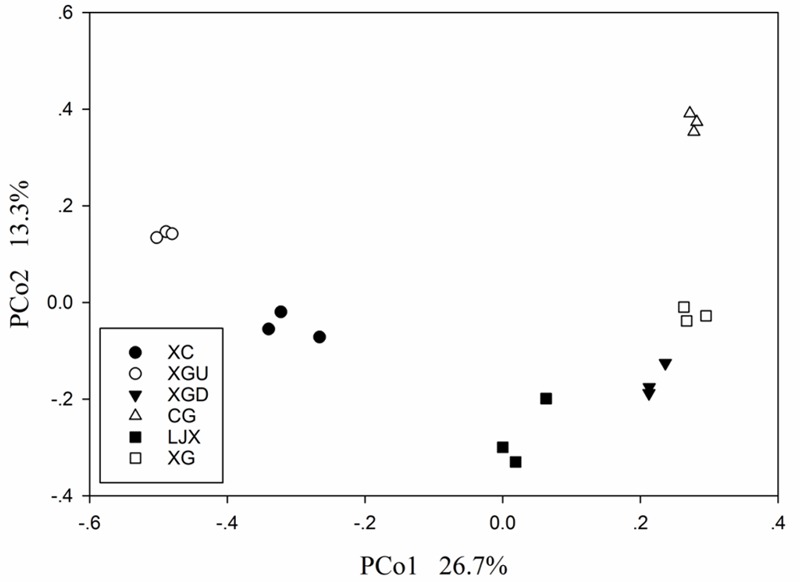
Principal coordinate analysis (PCoA) score plot of different riparian soil samples based on weighted UniFrac metrics. Two categories were clustered, including uncontaminated group – XC and XGU and contaminated group – XGD, LJX, XG, and CG.

Principal coordinate analysis was conducted to investigate the dissimilarities among the six sites. The first axis explained 26.7% variance of species and 13.3% was explained by the second axis (**Figure 4**). All samples formed two clusters as follows: uncontaminated group containing the XC and XGU samples and the contaminated group including both slightly contaminated and heavily contaminated samples. This result is consistent with the Cr pollution condition indicating that Cr may be the main factor altering microbial community structure.

### Correlation between the Microbial Communities and Environmental Variables

Soil organic matter and water content exhibited positive correlations with K, Cu, Cr, and Mn (*p* < 0.01) while K, Cu, Zn, and Cr showed positive correlations with Mn (*p* < 0.05). Additionally, Cr was significantly and positively correlated with K and Cu (**Table 2**). In general, the salt extractable part of heavy metals showed significant and high positive correlations with the total heavy metal concentrations (*p* < 0.01, Supplementary Table S3). This result indicated that SCr could reflect the total Cr contamination condition. Although Cr (VI) reduction ability presented significantly positive correlations with SCu, SCr, and microbial biomass (*p* < 0.01), the correlation coefficient with SCr was the highest, reaching up to 0.889. Microbial biomass also presented significant correlations with SK, SCu, and SCr (*p* < 0.05, Supplementary Table S3).

**Table 2:** Spearman’s correlation coefficients between environmental variables.

	SOM	Water content	pH	Mn	Cr	Zn	Cu	K	P
N	0.383	0.386	-0.155	0.436	0.347	0.208	0.743^∗∗^	-0.056	0.483^∗^
P	0.341	0.301	0.481^∗^	0.442	0.297	0.024	0.258	-0.132	
K	0.647^∗∗^	0.606^∗∗^	0.146	0.560^∗^	0.641^∗^	0.585^∗^	0.238		
Cu	0.664^∗∗^	0.746^∗∗^	0.035	0.480^∗^	0.651^∗∗^	0.255			
Zn	0.439	0.424	0.093	0.608^∗∗^	0.408				
Cr	0.928^∗∗^	0.928^∗∗^	0.266	0.725^∗∗^					
Mn	0.839^∗∗^	0.732^∗∗^	0.167						
pH	0.255	0.207							
Water content	0.926^∗∗^								

*^∗∗^Means the correlation is significant at the *p* < 0.01 level. ^∗^Means the correlation is significant at the *p* < 0.05 level.*

**FIGURE 5:**
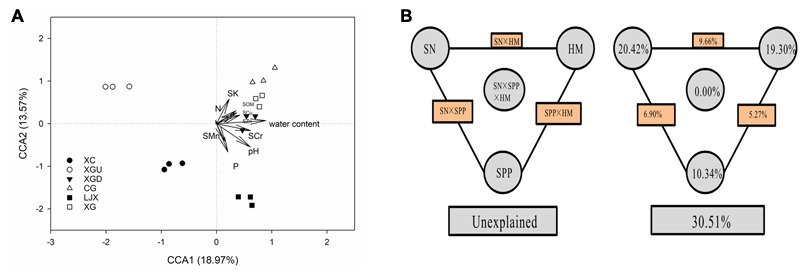
**(A)** Canonical correspondence analysis (CCA) of microbial community structure and measurable variables of different samples. Arrows indicate the direction and magnitude of measurable variables associated with microbial community structure. **(B)** Variation partition analysis (VPA) of the effects of heavy metals (HM: SCu, SZn, SCr, SMn), soil physical properties (SPP: pH, water content) and soil nutrient (SN: SK, N, P, and SOM) on microbial community structure. The edge represents the variation explained by each factor alone. The rectangles between each set of factors represent the interactions of the two factors, and the oval in the middle represents the interaction of all three factors.

Canonical correspondence analysis and VPA were performed to evaluate the relative contributions of different environmental variables to changes in the microbial community structure. Physicochemical characteristics of the riparian soils, including heavy metals (HM: SCu, SZn, SCr, SMn), soil physical properties (SPP: pH, water content), and soil nutrient (SN: SK, N, P, and SOM) were considered as the environmental parameters (**Figure 5A**). As illustrated from the results from CCA (*p* = 0.001), the two axes explained 18.97 and 13.57% of the microbial community differentiation, respectively. The CCA results showed that pH and water content largely shaped the microbial community even though pH values varied little among the different sites. As indicated, SCr, SCu, SZn, and SOM were also strongly linked to the microbial community correspondingly to the length of the vector. SMn, SK, P, and N had lower contribution to the structure of microbial communities. VPA was performed to further discern the possible relationship between microbial communities and environmental variables. The results of VPA showed that 69.49% of the variance in the microbial community structure could be explained by three major types of variables, i.e., HM, SPP, and SN, leaving 30.51% of the variation unexplained (ANOVA *p* = 0.001). SN alone only explained 20.42% of the variation and half of its contribution was shared with HM (9.66%). HM alone explained 19.30% of the variation, among which SCr, as the single factor, accounted for 5.80%. This result indicates that Cr contamination contributed a lot to the differences of microbial communities among those samples. SPP alone explained 10.34% of the variation. Moreover, significant interactive effects among the rest of the factors including interaction of SN with SPP (6.90%) and SPP with HM (5.27%) were observed, while no significant interaction of those three factors (0.00%) was shown in this study (**Figure 5B**).

Spearman’s correlation analysis at the phylum level (**Table 3**) showed that *Firmicutes* and *Bacteroidetes* displayed the highest positive correlation coefficients with SCr, whereas *Actinobacteria, Acidobacteria*, and *Crenarchaeota* presented negative correlations (*p* < 0.01). The correlation of SOM and water content with the relative abundances of dominant microbial communities was similar to that of SCr. SCu was negatively correlated with *Actinobacteria* and *Acidobacteria*. Unexpectedly, *Chloroflexi* had a positive correlation with SZn. In addition, *Actinobacteria* and *Euryarchaeota* exhibited negative correlations with pH (*p* < 0.05) although the pH value range varied little among the six sites.

**Table 3:** Spearman’s correlation coefficients between the relative abundance of the dominant microbes (at phylum and genus level, respectively) and environmental variables.

	N	P	SK	SCu	SZn	SCr	SMn	pH	Water content	SOM
***Proteobacteria***	0.344	-0.158	0.482*	0.333	0.154	0.207	-0.311	-0.211	0.143	0.214
*Devosia*	0.413	0.142	0.056	0.002	0.021	-0.433	0.010	-0.560*	-0.440	-0.470*
*Desulfobacter*	0.494*	0.382	0.488*	0.665**	0.343	0.910**	0.488*	0.073	0.905**	0.903**
*Hydrogenophaga*	-0.207	0.085	0.199	0.042	0.169	0.593**	0.568*	0.091	0.647**	0.643**
*Rhodoferax*	-0.076	-0.104	0.437	0.199	0.453	0.669**	0.162	0.119	0.736**	0.759**
*Thiobacillus*	0.068	-0.010	0.459	0.317	0.497*	0.706**	0.033	0.068	0.718**	0.766**
*Azovibrio*	0.252	0.297	0.387	0.376	0.318	0.757**	0.222	0.141	0.781**	0.856**
*Geobacter*	0.211	0.343	0.291	0.448	0.173	0.832**	0.480*	0.290	0.818**	0.888**
*Rhodobacter*	0.056	0.067	0.482*	0.320	0.347	0.764*	0.417	0.004	0.790**	0.792**
*Skermanella*	-0.020	0.061	-0.300	-0.380	0.160	-0.489*	0.162	-0.415	-0.454	-0.462
***Firmicutes***	0.202	0.353	0.247	0.362	0.129	0.791**	0.432	0.416	0.769**	0.820**
*Virgibacillus*	-0.022	0.129	-0.067	-0.257	0.290	-0.074	0.332	-0.237	0.099	0.045
*Clostridium*	0.207	0.349	0.247	0.487*	0.166	0.859**	0.330	0.447	0.801**	0.808**
*Proteiniclasticum*	0.133	0.199	0.340	0.312	0.113	0.696**	0.566*	-0.028	0.637**	0.644**
*PSB-M-3*	0.020	0.228	0.217	0.323	0.083	0.767**	0.525*	0.361	0.748**	0.753**
***Bacteroidetes***	-0.250	-0.211	0.364	0.183	0.046	0.635**	0.364	0.083	0.620**	0.604**
*Paludibacter*	0.443	0.373	0.405	0.627**	0.257	0.875**	0.588*	0.108	0.859**	0.859**
*Flavobacterium*	-0.206	-0.154	0.380	0.137	0.394	0.638**	0.215	0.102	0.699**	0.689**
***Actinobacteria***	-0.099	-0.086	-0.160	-0.474*	-0.090	-0.706**	-0.086	-0.575*	-0.645**	-0.668**
*Iamia*	0.190	0.193	-0.359	-0.207	0.016	-0.570*	-0.060	-0.229	-0.624**	-0.638**
*Arthrobacter*	0.026	0.004	-0.068	-0.416	0.016	-0.530*	-0.080	-0.526*	-0.528*	-0.477*
***Acidobacteria***	-0.379	-0.361	-0.297	-0.634**	-0.065	-0.765**	-0.366	-0.349	-0.752**	-0.695**
*Candidatus Solibacter*	-0.266	0.193	-0.316	-0.298	0.163	-0.095	-0.232	0.421	-0.047	-0.024
***Chloroflexi***	-0.265	-0.541*	0.162	-0.216	0.521*	-0.208	-0.236	-0.367	-0.102	-0.156
*WCHB1-05*	0.110	-0.037	0.591**	0.404	0.368	0.774**	0.125	0.045	0.803**	0.828**
*Anaerolinea*	-0.309	-0.233	0.328	0.063	0.237	0.573*	0.075	0.118	0.579*	0.622**
***Euryarchaeota***	-0.069	-0.154	0.057	-0.028	0.036	-0.128	-0.003	-0.549*	-0.242	-0.282
*Methanosarcina*	0.168	0.194	0.338	0.333	0.220	0.604**	0.793**	-0.264	0.634**	0.590*
***Crenarchaeota***	-0.160	0.072	-0.342	-0.416	-0.100	-0.617**	-0.152	-0.269	-0.596**	-0.577*
*Candidatus Nitrososphaera*	-0.270	-0.025	-0.401	-0.517*	-0.269	-0.736**	0.124	-0.414	-0.674**	-0.717**

*^∗∗^Means the correlation is significant at the *p* < 0.01 level. ^∗^Means the correlation is significant at the *p* < 0.05 level.*

The relative abundances of 20 different microbial genera correlated with SCr, water content or SOM significantly (*p* < 0.01) and 18 of them, such as *Desulfobacter, Azovibrio*, and *Geobacter*, showed significant correlation with both SCr, water content, and SOM simultaneously. However, *Skermanella* was the only genus which was significantly correlated with SCr not with SOM or water content. This result implied that SCr, water content and SOM have comprehensive impacts on the microbial communities rather than individual impacts. SCu and SMn were significantly (*p* < 0.05) correlated with four and seven genera, respectively. In addition, pH was only significantly correlated with two genera at *p* < 0.05. In general, only a few genera showed significant correlation with N, P, SK, and SZn. This result indicates that N, P, SK, SCu, SZn, SMn, and pH have minor influence on the abundances of microbial communities as compared to the influences of water content, SCr, and SOM.

## Discussion

The Yellow River as one of the most important water resources for agriculture, industry, and population in northern China and its heavy metal contamination condition is a complex phenomenon (Yu et al., 2009). Previous studies have shown that the heavy metal concentrations (in mg kg^-1^ dry weight) in 2008 ranged from 89.80–201.88 (Zn), 41.49–128.30 (Cr), 29.72–102.22 (Cu), and 773.23–1459.699 (Mn) (Liu et al., 2009). However, in our study, only Zn, Cr, and Mn showed higher contamination concentration (in mg kg^-1^ dry weight) and ranged from 57.64–342.55 (Zn), 83.83–506.58 (Cr), and 5056.67–10753.33 (Mn) while Cu showed a lower concentration, reaching up to 3.63–25.58 mg kg^-1^. Since Cu concentration in both researches was low, it implied that Cu is not the main pollutant in this area. Previous studies have shown that Cr contamination is severe in the Pearl River estuary, South China, varying from 91.42 to 125.21 mg kg^-1^, whereas Cr concentration in surface sediment from 59 stations ranged from 36.9 to 173 mg kg^-1^ in Yangtze River (Zhang et al., 2009; Bai et al., 2011b). This indicated that Cr is a common pollutant in the river and Cr concentration in Gansu industrial reach of the Yellow River is much higher than other aquatic systems reported in China. Thus, this study focused on the Cr contamination.

The qRT-PCR results showed that the expression of the Cr (VI) remediation genes *chrA* and *yieF* of the XG and CG samples, was significantly (*p* < 0.05) up-regulated after 30 min Cr (VI) treatment. Induction of the two genes by Cr was always the case in Cr reducing bacteria such as *Lysinibacillus fusiformis ZC1* (He et al., 2011). Further, copies of *chrA* and *yieF* did not change after incubation with Cr (VI) in the lab for 35 days (**Figures 2C,D**). This result indicated that the adaption of microbial community may only depend on the expression level changes of heavy metal resistance genes. Thus, analysis of the function of microbial community should be focused on the both DNA and RNA of microbes in the environment. Metagenomic sequencing data also showed that the microbial community in ground water contaminated long-term with heavy metals possessed heavy metal resistance genes such as *chrAB* chromate efflux, *CzcABC* efflux, and *mer* operon (Yu et al., 2016). Although metagenomic sequencing provided a comprehensive way to understand microbial community function, it has a very limited function in revealing microbially expressed functions or microbial community activities (Wang W.-L et al., 2015). Therefore, compared to high-throughput metagenomic methods, the phenotype test is a straightforward way to evaluate heavy metal contamination conditions. Microbial Cr (VI) remediation ability is related to Cr remediation related genes and proteins in microorganisms (Pradhan et al., 2016). Although *chrA* and *yieF* were not found in the XGD and LJX samples, the microbial communities of these three sites also exhibited Cr (VI) reduction ability. This may be due to the function of other Cr (VI) reductases, such as SOD, catalase, NemA, and LpDH (Thatoi et al., 2014; Pradhan et al., 2016). Although the riparian soil samples were incubated in the laboratory, the copies of *chrA* and *yieF* did not change after incubation. This result indicated that microbial community function was not affected in our lab incubation conditions.

Spearman’s correlation analysis showed that SOM exhibited a positive correlation with K, Cu, Cr, and Mn (*p* < 0.01) (**Table 2**) which is consistent with previous studies that highlight that SOM can act as a major sink for heavy metals due to its strong complexing capacity (Zayre et al., 2006). Previous reports also showed that SOM can keep Cu mobility low in soil by chemisorptions and accelerated the reduction of toxic and mobile Cr (VI) to stable Cr (III) (Bai et al., 2011b). However, Zn did not significantly correlate with SOM suggesting that the SOM might be mainly imported by Cu, Cr, and Mn in the study area. A previous study has also depicted that heavy metals might not always be immobilized by SOM under certain conditions of high inputs of anthropogenic interfere (Kumpiene et al., 2008). K, Cu, Cr, and Mn also showed significant correlations with water content which also correlated with heavy metals significantly (**Table 2**). This result is in agreement with previous studies that soil water capacity increased with SOM content (Hudson, 1994) and thus, metals exhibit a correlation with water content. Significantly positive relationship between Mn and K, Cu, Zn, or Cr was identified which indicates that these metals may come from the same source or similar transportation mechanisms and get accumulated in the soils (Qiu, 2010). Additionally, the salt exchangeable part of metals was measured to analyze the correlation with microbes since the bioavailability of total metals can be strongly influenced by other environmental factors such as pH (Zhu et al., 2013). The salt extractable parts of SCu, SZn, and SCr exhibited high significant correlations with the total concentrations of Cu, Zn, and Cr (*p* < 0.01, Supplementary Table S3) which indicated that the analysis of the salt extractable part of these heavy metals with microbes could reflect the relationship between their total heavy metal condition and the microbial community. Additionally, the total Cr concentrations did show significant and high positive correlations with the Cr (VI) reduction ability of microbial community (*p* < 0.01, Supplementary Table S3) which implicated that Cr (VI) reduction ability of the microbial community could reflect the total Cr concentration condition in this area. Moreover, Cr (VI) reduction ability presented significantly positive correlations with microbial biomass (*p* < 0.01) which indicated that there may be some microbes in soil could be indicators of Cr. Thus, we speculate that Cr might have a great effect on microbial community structure and function in this area.

Although the function of microbial communities could also separate contaminated samples from uncontaminated samples, microbial community structure analysis is necessary for revealing the instant microbial compositions and Cr indicators. PCoA showed microbial communities in the six sites were divided into two groups, the uncontaminated group and the contaminated group. This is similar with a previous study in that PCoA of microbial community structure in sediments collected from four sites revealed different levels of heavy metal contamination along the Xiangjiang River (Zhu et al., 2013).

MiSeq sequencing and Spearman’s correlation analysis were performed to analyze the structure of the microbial community in the riparian soil samples of the six sites. Previous reports state that *Proteobacteria* was the most abundant phylum, both in highly Cr-contaminated soil samples and uncontaminated soil samples of a tributary of the Alviela River, reaching 66 and 47%, respectively (Desai et al., 2009). This is consistent with our study where *Proteobacteria* was the most abundant phylum in samples from all six sites and did not show significant correlation with Cr (**Figure 2A** and **Table 3**). *Firmicutes* and *Bacteroidetes* in the contaminated group were richer than in the uncontaminated group and positively correlated with SCr (**Figure 3A** and **Table 3**). Numerous studies have confirmed that *Firmicutes* and *Bacteroidetes* are the main phyla in the Cr contaminated environment (Miao et al., 2015; Zhang et al., 2017). The relative abundance of *Actinobacteria* ranged from 1.59 to 19.89% for the six sites, showing a negative correlation with SCr (**Figure 3A** and **Table 3**). This is not consistent with a previous study which reported that after 28 days of Cr (VI) incubation, based on the DGGE analysis, *Actinobacteria* became one of the most abundant phyla in agricultural soils collected from the Mediterranean area of Santa Bàrbara, Tarragona (Branco et al., 2005). The difference in *Actinobacteria* relative abundance may be due to the diverse methods used. Further, *Iamia* and *Arthrobacter*, as the most abundant genera of *Actinobacteria*, were sensitive to Cr in our study which are in line with a previous study where *Arthrobacter* was correlated with heavy metals (Ellis et al., 2003). Almost all samples contained similar amounts of *Chloroflexi* except for the CG site as *Chloroflexi* is a common phyla in aquatic ecosystems (Yamada and Sekiguchi, 2009). MiSeq sequencing data showed that the percentages of *Desulfobacter, Hydrogenophaga, Rhodoferax, Thiobacillus, Azovibrio, Geobacter, Rhodobacter, Clostridium, Proteiniclasticum, PSB-M-3, Paludibacter, Flavobacterium, WCHB1-05, Anaerolinea*, and *Methanosarcina* in the contaminated group were higher than those in the uncontaminated group (**Figure 3B**). These 15 genera showed a positive correlation with SCr (**Table 3**) which indicated they may have Cr remediation potential under diverse Cr pressure and could be regarded as indicators for Cr pollution. This result is in agreement with previous studies. *Desulfobacter, Hydrogenophaga*, and *Rhodobacter* were found to be correlated with trace heavy metals (Martins et al., 2009; Bier et al., 2015). As revealed by genome sequencing, *Rhodoferax* was reported as a well-adapted bacteria that could deal with many environmental assaults including heavy metals, organic contamination, and oxidative stress (Risso et al., 2009). *Thiobacillus* could remove 100% of the Cr from tannery sludge after 8 days via bioleaching technology (Zhou et al., 2004). *Geobacter* and *Clostridium* are known to be involved in multiple metal reduction processes including Cr (VI), U (VI), and Fe (III) and perform *in situ* bioremediation at high levels of Cr contamination (Yu et al., 2016). *Proteiniclasticum* (6.66%) is one of the most abundant genera in the sediments of the Xiangjiang River, China, where Cr concentrations are 591–805 times of the national standard value (Zhu et al., 2013). *Anaerolinea* and *Methanosarcina* have also been reported in Cr contaminated environment (Somenahally et al., 2013; Yu et al., 2016). Although *Azovibri, PSB-M-3, Flavobacterium*, and *WCHB1-05* have not been reported in Cr-contaminated environments, they have been found under the pressure of other contaminants such as linear alkylbenzenesulfonate, nonylphenol, Cd, and petroleum (Allen et al., 2007; Kuffner et al., 2008; Rajbanshi, 2009; Macedo et al., 2015; Wang Z. et al., 2015). In contrast, the uncontaminated group contained different dominant bacteria such as *Skermanella, Iamia, Arthrobacter*, and *Candidatus Nitrososphaera* which were negatively correlated with Cr (**Table 3**). The microbial community structures in the riparian soil samples of these six sites are generally shaped by the severity of Cr contamination and some of the details from the microbial community data could be Cr indicators in the Gansu industrial reach of the Yellow River.

In this study, the combined analysis of microbial community structure and function provided a comprehensive insight into evaluating the severity of heavy metal contamination situations. Although the severity of Cr contamination could be assessed depending on microbial community function alone, microbial community structure analysis is also indispensable. Through the analysis of microbial community structure, not only the instant microbial composition was revealed but also the Cr indicators could be identified. In conclusion, combined microbial community composition and phenotypic function identification are potentially valuable for evaluating the severity of Cr contamination in the Gansu industrial reach of the Yellow River. Although bioinformatics analysis and functional verification showed Cr remediating microbes in this study, their molecular mechanisms should be clarified. Thus, further research should be aimed at the genera positively correlated with Cr and elucidation of their mechanisms related to genes which adapted to Cr contamination via metagenomic and metatranscriptomic approaches.

## Author Contributions

YP did most of the experiments, analyzed data, and contributed to writing and revising the manuscript. ZY extracted DNA of soil samples and contributed to analyzing data, and revising the manuscript. JJ helped with detecting physicochemical properties of soils. AK contributed to revising the manuscript. XL provided overall directions and contributed to revising the manuscript. All authors approved submission of this manuscript to Frontiers in Microbiology.

## Conflict of Interest Statement

The authors declare that the research was conducted in the absence of any commercial or financial relationships that could be construed as a potential conflict of interest.
