# Supplementary material for: Microbial Community Structure and Function Indicate the Severity of Chromium Contamination of the Yellow River
Source: Front Microbiol. 2018 Jan 25;9:38. doi: 10.3389/fmicb.2018.00038 (PMC5810299; doi:10.3389/fmicb.2018.00038)
Supplement: Supplementary file 1 [file Table_1.DOC]

**Table S1** Primers used in qRT-PCR.

| Genes | Primer | Sequence (5' to3') | Description |
| --- | --- | --- | --- |
| *16S rDNA* | 357F | CTCCTACGGGAGGCAGCAG | house-keeping gene |
|  | 519R | GWATTACCGCGGCKGCTG |  |
| *chrA* | chrAF | GGCTCAACGACAAGCAGTTC | chromate transporter |
|  | chrAR | ACGACCGTGAACAGATAGCAC |  |
| *yief* | yiefF | GGCAGGTAAACCGGTATTGAT | chromate reductase |
|  | yiefR | GGTTTGCGGATCAACTTTGT |  |
