# Supplementary material for: Microbial Community Structure and Function Indicate the Severity of Chromium Contamination of the Yellow River
Source: Front Microbiol. 2018 Jan 25;9:38. doi: 10.3389/fmicb.2018.00038 (PMC5810299; doi:10.3389/fmicb.2018.00038)
Supplement: Supplementary file 2 [file Table_2.DOC]

**Table S2** Microbial diversity indices calculated based on a cutoff of 97% similarity of 16S rRNA gene sequences. All data are presented as means ± standard deviations (n=3). Values with different letters in a column mean significant differences at *p*<0.05 as determined by Tukey’s test.

| Sample | Chao1 estimator of richness | Observed species | Shannon's diversity index | Simpson's diversity index |
| --- | --- | --- | --- | --- |
| XC | 5263 ± 1284^ab^ | 2943 ± 515^a^ | 10.320 ± 0.598^a^ | 0.997 ± 0.002^a^ |
| XGU | 3869 ± 117^ab^ | 2287 ± 85^ab^ | 9.470 ± 0.259^a^ | 0.992 ± 0.003^a^ |
| LJX | 3381 ± 461^b^ | 1934 ± 416^b^ | 8.453 ± 1.544^a^ | 0.974 ± 0.032^a^ |
| XGD | 5596 ± 600^a^ | 2641 ± 200^ab^ | 9.520 ± 0.173^a^ | 0.991 ± 0.002^a^ |
| CG | 4590 ± 290^ab^ | 2391 ± 122^ab^ | 9.807 ± 0.136^a^ | 0.997 ± 0.001^a^ |
| XG | 5225 ± 279^ab^ | 2666 ± 138^ab^ | 9.940 ± 0.035^a^ | 0.997 ± 0.001^a^ |
