# Supplementary material for: Microbial Community Structure and Function Indicate the Severity of Chromium Contamination of the Yellow River
Source: Front Microbiol. 2018 Jan 25;9:38. doi: 10.3389/fmicb.2018.00038 (PMC5810299; doi:10.3389/fmicb.2018.00038)
Supplement: Supplementary file 3 [file Table_3.DOC]

**Table S3** Spearman’s correlation coefficients between environmental variables and biofactors. **means the correlation is significant at the *p* < 0.01 level. *means the correlation is significant at the *p* < 0.05 level.

|  | Chromate reduction ability | Microbial biomass | K | Cu | Zn | Cr | Mn |
| --- | --- | --- | --- | --- | --- | --- | --- |
| SK | 0.455 | **0.531*** | **0.517*** |  |  |  |  |
| SCu | **0.702**** | **0.750*** |  | **0.979**** |  |  |  |
| SZn | 0.410 | 0.203 |  |  | **0.899**** |  |  |
| SCr | **0.889**** | **0.652**** |  |  |  | **0.921**** |  |
| SMn | 0.107 | 0.459 |  |  |  |  | 0.437 |
| Chromate  reduction ability | | |  |  |  | **0.890**** |  |
| Microbial biomass | **0.489**** |  |  |  |  |  |  |
