# Supplementary figures and images for: Microbial Community Structure and Function Indicate the Severity of Chromium Contamination of the Yellow River
Source: Front Microbiol. 2018 Jan 25;9:38. doi: 10.3389/fmicb.2018.00038 (PMC5810299; doi:10.3389/fmicb.2018.00038)

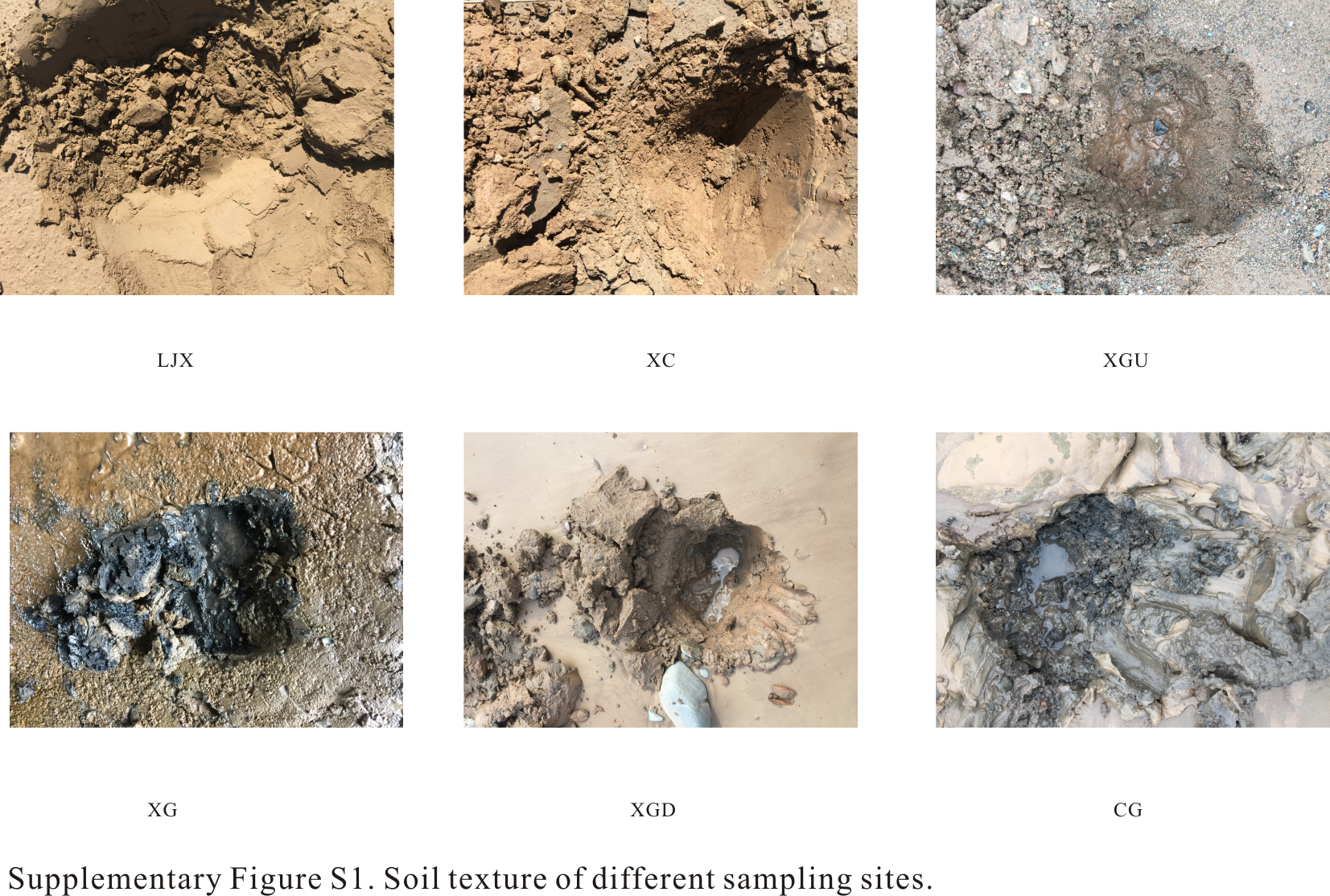

Supplement: Supplementary file 4 [file Image_1.tif]

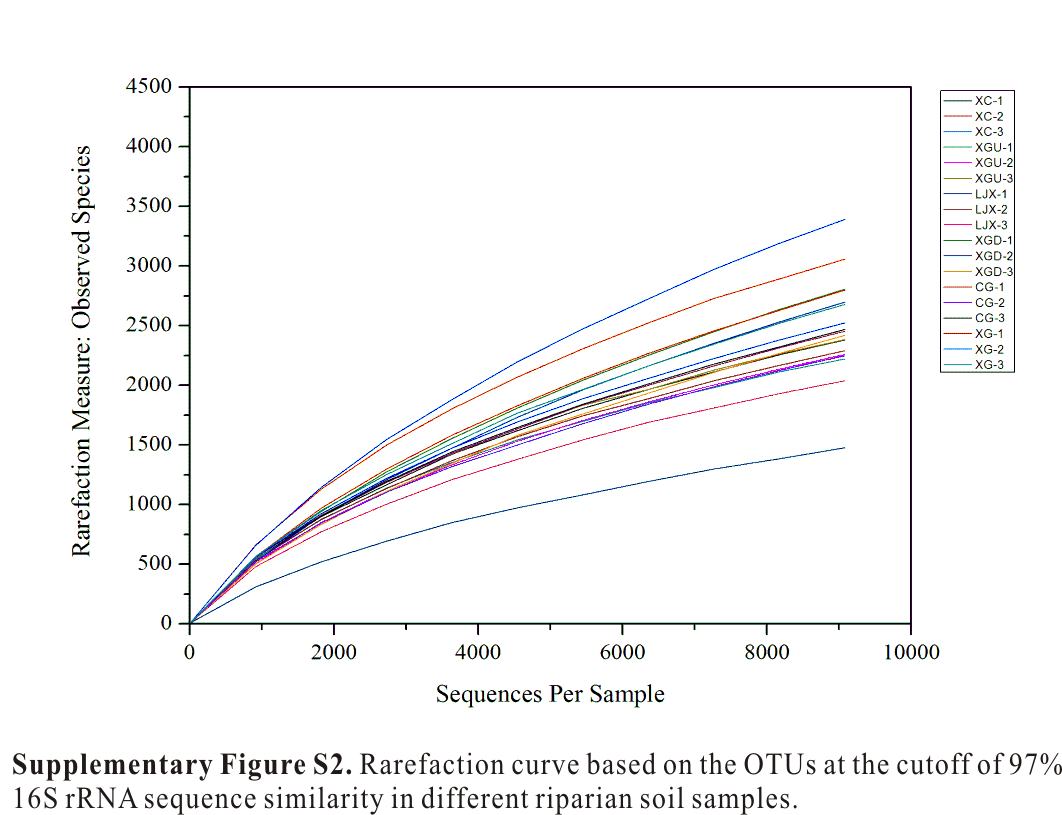

Supplement: Supplementary file 5 [file Image_2.tif]
